# “Ruffled border” formation on a CaP-free substrate: A first step towards osteoclast-recruiting bone-grafts materials able to re-establish bone turn-over

**DOI:** 10.1007/s10856-018-6046-4

**Published:** 2018-03-21

**Authors:** Antonio Merolli, Stephanie Fung, N. Sanjeeva Murthy, E. Thomas Pashuck, Yong Mao, Xiaohuan Wu, Joseph A. M. Steele, Daniel Martin, Prabhas V. Moghe, Timothy Bromage, Joachim Kohn

**Affiliations:** 1 New Jersey Center for Biomaterials, Rutgers – The State University of New Jersey, New Brunswick, NJ 08901 USA; 2 Universita Cattolica del Sacro Cuore, Clinica Ortopedica, Rome, Italy; 3 High Resolution Microscopy, Biomedical Engineering, Rutgers – The State University of New Jersey, New Brunswick, NJ 08901 USA; 4 Hard Tissue Research Unit. Department of Biomaterials and Biomimetics, New York University College of Dentistry, New York, NY 10010 USA

## Abstract

Osteoclasts are large multinucleated giant cells that actively resorb bone during the physiological bone turnover (BTO), which is the continuous cycle of bone resorption (by osteoclasts) followed by new bone formation (by osteoblasts). Osteoclasts secrete chemotactic signals to recruit cells for regeneration of vasculature and bone. We hypothesize that a biomaterial that attracts osteoclasts and re-establishes BTO will induce a better healing response than currently used bone graft materials. While the majority of bone regeneration efforts have focused on maximizing bone deposition, the novelty in this approach is the focus on stimulating osteoclastic resorption as the starter for BTO and its concurrent new vascularized bone formation. A biodegradable tyrosine-derived polycarbonate, E1001(1k), was chosen as the polymer base due to its ability to support bone regeneration *in vivo*. The polymer was functionalized with a RGD peptide or collagen I, or blended with β-tricalcium phosphate. Osteoclast attachment and early stages of active resorption were observed on all substrates. The transparency of E1001(1k) in combination with high resolution confocal imaging enabled visualization of morphological features of osteoclast activation such as the formation of the “actin ring” and the “ruffled border”, which previously required destructive forms of imaging such as transmission electron microscopy. The significance of these results is twofold: (1) E1001(1k) is suitable for osteoclast attachment and supports osteoclast maturation, making it a base polymer that can be further modified to optimize stimulation of BTO and (2) the transparency of this polymer makes it a suitable analytical tool for studying osteoclast behavior.

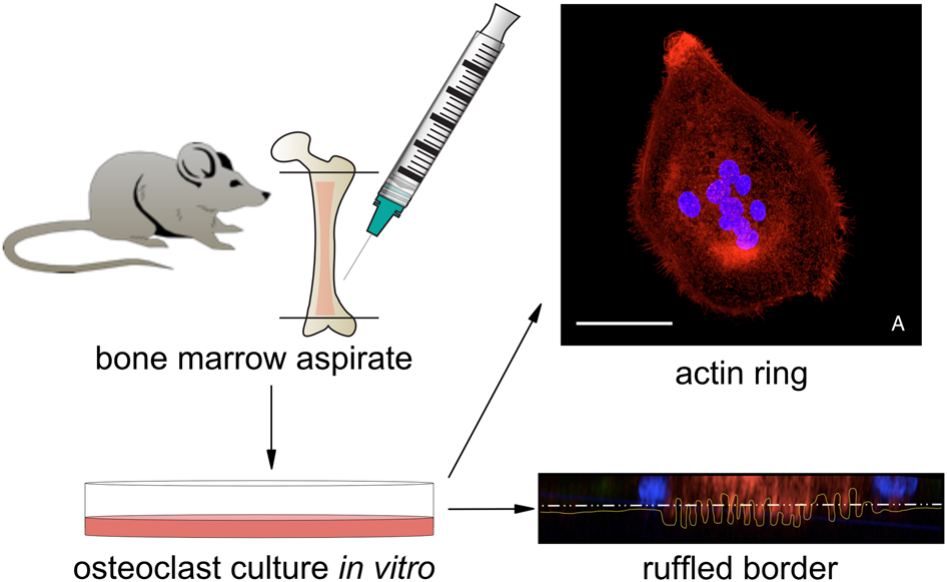

## Introduction

Our long-term goal is to develop a new strategy to design a clinically-effective artificial bone-graft that is able to promote bone regeneration in cases of major bone loss and non-union.

There is a need for an alternative option to current artificial bone-grafts because they have not provided an effective solution despite more than two decades of basic and applied research. Current artificial bone-grafts promote the early formation of bone, but they often fail to complete the healing process towards remodeling vascularized new bone [1, 2]. The combination of an osteogenic environment with an effective vascular network is what is needed for the treatment of large bone defects [3, 4], but this may require a paradigm change in the functional characteristics of biomaterials for artificial bone graft [3]. Our strategy is based on the development of a biomaterial that is able to re-establish the physiological bone turnover (BTO), which is the continuous cycle of bone resorption by osteoclasts followed by new bone formation by osteoblasts [5].

**Fig. 1:**
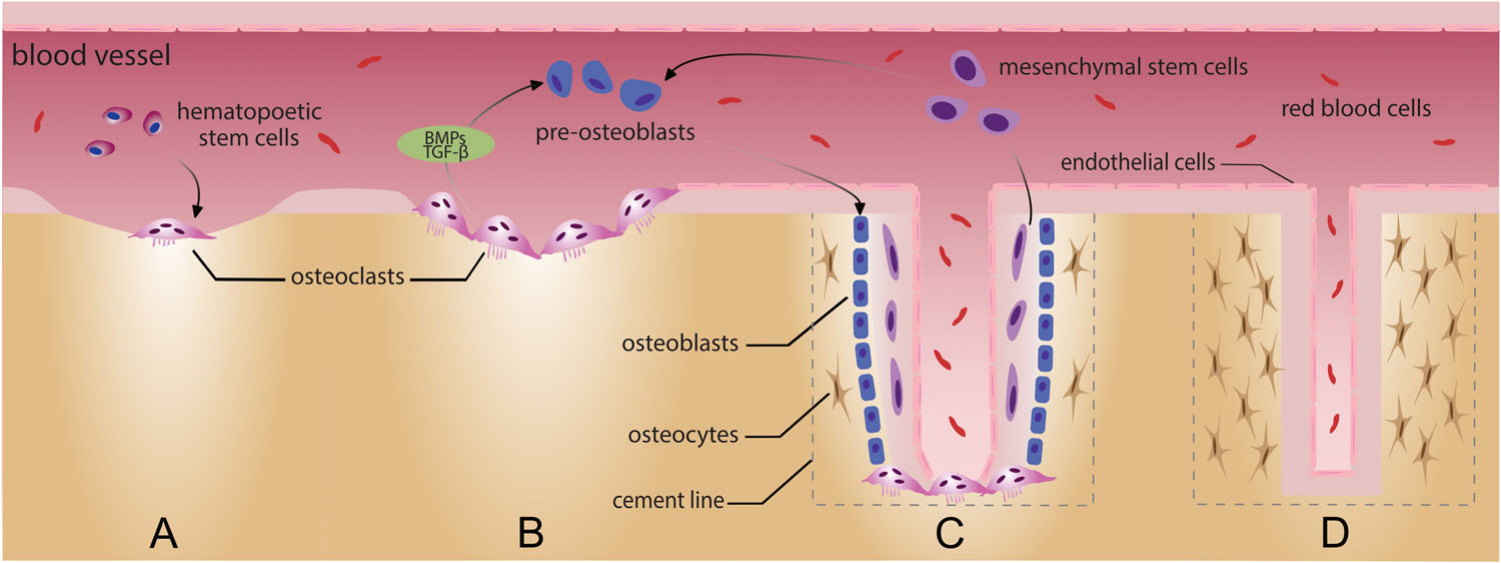
**a** OSC are large, multinucleated giant cells derived from blood-stream mononuclear precursors of the hematopoietic lineage which are recruited at the site of old bone resorption. **b** OSC initiate BTO by digging a tunnel into the old bone and secreting guidance clues for osteoblasts and blood vessels which follow behind. **c** Osteoblasts will then form new vascularized bone in the void and **d** will entrap themselves, becoming osteocytes. This process takes about 4 months in humans

Bone is produced in embryo from mesenchymal or cartilaginous templates [6]. After this first *de novo* bone formation, a lifelong cycle of osteoclastic resorption of old bone followed by osteoblastic deposition of new bone takes place. This continuous regenerative cycle is such that the adult skeleton may be entirely replaced every ten years [2]. The first step in BTO is the recruitment of osteoclast precursors from blood-stream mononuclear cells of the hematopoietic lineage. These cells fuse to generate mature osteoclasts (OSC) that digest the old bone (or the biomaterial, in our case). Osteoclasts initiate BTO by digging a trench (in trabecular bone) or a tunnel (in cortical bone) thus providing the space and the chemotrophic factors which act as guidance clues for osteoblasts and blood vessels which follow behind. Osteoblasts will, then, form the new vascularized bone (Fig. 1) [6–11].

**Fig. 2:**
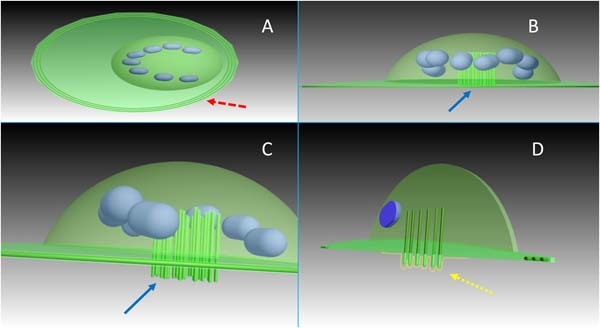
**a** OSC create a sealed zone inside their perimeter: the “actin ring” (red arrow). **b** Pillars of actin (blue arrow) are found at the center of the OSC (giving the nuclei an horse-shoe arrangement as seen in (**a**)). **c** A kind of “drilling complex” (blue arrow) is deputed to resorb the substrate, producing a typical round resorption pit (or lacuna). **d** A histological section taken at this level will show the characteristic expansion of the membrane at the interface with the substrate (yellow arrow), in the fashion of a “ruffled border” (dotted line) (color figure online)

**Fig. 3:**
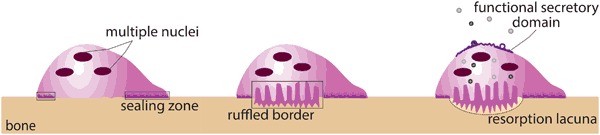
OSC act in three functional steps: they seal off a circumferential area on the substrate by forming an actin ring (left); then they expand the surface area of their membrane which faces the substrate, creating a ruffled border that can be seen in histological sections; a resorption lacuna (pit) can be found below this area (center). Finally, digested collagen and solubilized minerals are transported in vesicles from the resorption lacuna towards the functional secretory domain, on the opposite site (right)

Osteoclasts are large, multinucleated giant cells (MNGC) that resorb bone by creating a sealed zone inside their perimeter where a circumscribed drop in pH occurs and where there is the release of specific collagenases. The low pH facilitates the dissolution of the bone mineral phase, exposing slender needle-shaped undigestable hydroxyapatite crystals [9]. Collagenases, such as cathepsin K, digest the organic phase of the bone (mostly collagen type I). A round osteoclastic “lacuna” (pit) results on bone surface from this drilling resorption process (Fig. 2). Multiple lacunae may coalesce in a long trench or in a tunnel as the process continues [10]. Osteoclasts act in three functional steps which are generally described as a sequence, but once the resorption process has been established can be observed simultaneously (Fig. 3) [6, 8]. *Step 1: attachment*. OSC seal off a circumferential area on the substrate by forming an “actin ring” (AR); resorption will take place in this area. Attachment is a dynamic process and OSC can move along a surface, still keeping the resorption area effectively sealed by the AR [8]. *Step 2: resorption*. OSC expand the surface area of their part of membrane which interfaces with the substrate, creating the “ruffled border” (RB) seen in histological sections; a resorption lacuna (pit) is found below this area. The sealing zone defined by the AR protects the adjacent cells from the acidic environment present at the RB [8]. *Step 3: vesicular trafficking*. OSC transport digested collagen and solubilized minerals in vesicles which are processed and “transcytosised” from the resorption lacuna towards the opposite side of the OSC, where they are secreted extracellular through an area called “functional secretory domain” (FSD) [6, 9]. The content of this secretion contains factors able to recruit osteoblasts and blood vessels into the tunnel [6, 11].

The *in vitro* culture on the flat and undigestable surfaces of poly-styrene plates documents that, after the fusion from mononuclear precursors, OSC form an AR and prepare to form a RB (“polarized osteoclasts”) [6, 12]. A substrate which allows the drilling machinery to create the resorption pit is required to progress from the “polarized osteoclast” to the “resorbing osteoclast” [12] which exhibit a RB and can be studied *in vitro* only disk slices of bone, dentin or calcium-phosphate (CaP) rich substrates [13, 14].

We hypothesize that a biomaterial that attracts osteoclasts, and/or can be effectively resorbed by them, will re-establish the physiological BTO which leads to newly formed vascularized bone. Therefore, such a biomaterial may elicit a better clinical efficacy in comparison with current bone graft materials as they rarely achieve the new vascularization and complete filling of the bone loss. Our ultimate clinical goal is the complete replacement of the new bone graft material by fully vascularized endogenous bone.

In this first study, we have been able to demonstrate how the early two steps in osteoclast activity, namely the formation of the “actin ring” and the formation of the “ruffled border”, can occur by using the CaP-free tyrosine-derived polycarbonate (E1001(1k)). E1001(1k) has shown promise *in vivo* where the polymer-bone interface exhibited morphological signs of integration and remodeling [15, 16]. We investigated the ability of E1001(1k) to support osteoclast recruitment and activation *in vitro*, alone or in blends with RGD polypeptide, collagen type I and β-TCP (beta-tricalcium phosphate), factors involved in steps 1, 2 and 3 respectively. Cultures on hydroxyapatite and on polystyrene served as controls. The formation of the RB was visualized by immunofluorescent staining and confocal microscopy.

We are aware that an *in vitro* evaluation has intrinsic limitations to incorporate all of the factors that affect cell behavior *in vivo*. However, *in vitro* methods may allow the study of the very early events in osteoclast activity, namely the “actin ring” and the “ruffled border” formation, in an easy-to-access and reproducible way. So, we see the *in vitro* analysis of osteoclast behavior as a necessary first step in the development of a future BTO-promoting, osteoclast-recruiting material.

## Materials and methods

### Experimental design

#### Selection and differentiation of OSC on polystyrene

To test our ability to obtain well-defined OSCs we established protocols to select macrophages from the bone marrow aspirate of the Sprague-Dawley rat by optimizing published protocols [17]. These macrophages were then stimulated to fuse into MNGC, which possess the morphological characteristics of osteoclasts (like a periphery of filopodia, a circular or horse-shoe arrangement of nuclei, etc.). We used the anti-RANK (receptor activator of nuclear factor-kappaB) antibody to mark specifically our MNGC as “osteoclasts”.

#### Attachment of OSC on different substrates

To test our ability to culture OSC on different substrates we seeded OSC on standard polystyrene plates, a substrate that they cannot digest. Then we seeded OSC on 1 mm thick hydroxyapatite discs (a standard artificial substrate to culture OSC, alternative to natural bone or dentin). Finally, we seeded OSC on the candidate polymer E1001(1k), alone or in three blends with RGD peptide, collagen type I and β-TCP.

#### Imaging of the “actin ring” and the “ruffled border”

To test the possibility to detect and visualize the early phases of OSC activity (namely AR and RB formation) with light transmitted through the substrate we used inverted fluorescence microscopy and laser confocal microscopy.

Transmitted light microscopy is an unusual modality in imaging OSC culture as bone, dentin or CaP-rich substrates have a degree of opacity. However, it can provide a direct visualization of the OSC-substrate interface.

### Substrate preparation

Solvent casting was used to prepare films of E1001(1k) alone and in combination with RGD, collagen type I and β-TCP. Before that, samples of E1001(1k) alone and E1001(1k) in combination with RGD were also prepared by spin-coating the polymer solutions onto glass coverslips. Solvent casting was adopted as a technique of choice after it was demonstrated that spin-coating will enable OSC attachment but will not produce films thick enough to be resorbed by OSC.

#### Spin coating technique

A 2% (w/v) solution of the polymer was prepared in 1:1 mixture of dichloromethane (DCM) and tetrahydrofuran (THF). The samples were spin coated under dry condition (8% RH) on a Headway Research Inc. (Garland, TX) spin coater. About 70 microliters of solution was placed on each cover slip, and spin coated at 3000 rpm for 30 s. The thickness of the film, as estimated from similar samples using quartz crystal microbalance, was ~100 nm.

#### Solvent casting technique

E1001(1k), RGD-modified E1001(1k), and E1001(1k)+ collagen type I films were prepared by dissolving 200 mg of the polymer in a 5 mL DCM. E1001(1k)-β-TCP was prepared by dissolving 127.5 mg of E1001(1k) in 5 ml of DCM. Films were cut into circles of 10 mm in diameter using a biopsy punch. Cut films fit into the bottom of 48-well tissue culture plates and had a thickness of about 25–45 microns.

##### RGD-modified E1001(1k)

The modified polymer was obtained by coupling RGD to E1001(1k) using a maleimide reaction with cysteine on the peptide sequence, produced by solid phase peptide synthesis and purified by HPLC. A GRGDSP peptide was synthesized using solid phase peptide synthesis. All amide couplings were done using *O*-(6-Chlorobenzotriazol-1-yl)-*N*,*N*,*N*′,*N*′-tetramethyluronium hexafluorophosphate (HCTU), unless otherwise noted. For each coupling the amino acid, HCTU and diisopropylethylamine (DIPEA) were added in a 4:4:6 ratio to peptide. After successful coupling the Fmoc group was removed by washing the resin with 20% piperidine in dimethylformamide (DMF) twice for 5 min. A ninhydrin test was performed to check for a positive result. For the back bone peptide the N-terminus was capped upon completion of the amino acid couplings. The peptide was then cleaved from the resin in 95% trifluouroacetic acid (TFA) (Sigma-Aldrich) and 2.5% triisopropylsilane (TIS). The cleavage solution was emptied into a round bottomed flask and washed 2 × with dichloromethane (DCM). The solution was removed using rotary evaporation, and then the product was precipitated in diethyl ether. This molecule was then dried under vacuum and dissolved in water. It was purified by high pressure liquid chromatography (HPLC) under acidic conditions. A gradient was run from 95% water/20% acetonitrile to 95% ACN/5% water with 0.1% TFA at 10 mLs/minute. Upon purification, the acetonitrile was removed through rotary evaporation, the solution was frozen and ice removed with lyophilization. 320 mg of E1001(1k) was dissolved into 10 mLs of DMSO and activated with 33.6 mg of HCTU and 22.4 mg of DIPEA in a round bottomed flask. After 5 min of activation 50 mg of the GRGDSP peptide was added to the mixture and it was stirred overnight. The next day the solution was precipitated in isopropanol, dissolved in DCM and reprecipitated in isopropanol. The polymer-peptide conjugate was placed under vacuum and conjugation was quantified using NMR.

##### E1001(1k)-collagen type I

The modified polymer was prepared by placing an E1001(1k) film in MES buffer (pH 4.5) and 50 milligrams of N-(3-Dimethylaminopropyl)-N′-ethylcarbodiimide hydrochloride (EDC) with 25 mg of N-Hydroxysuccinimide (NHS) for 5 min. The sheet was removed from the MES solution after 5 min, washed briefly with the MES solution, and then added to a fresh solution of MES buffer with 10 mg of collagen. The collagen was reacted with the film over night, after which it was washed 3× with PBS and then dried under vacuum.

##### E1001(1k)-β-TCP

Composite polymer film was prepared by blending 15 w/v% beta-TCP (Berkeley Advanced Biomaterials Inc, Berkeley CA) with a 25.5 w/v% solution of E1001(1k) in DCM. Films were cast in Teflon dish and allowed to dry at a constant vapor pressure overnight and then dried under vacuum at 45 C for 18 h.

#### Sterilization

Films were sterilized by UV for 2 h; the UV lamp was a Sylvania G36T5/SP Germicidal UV-C 39 W Fluorescent Lamp emitting at 254 nm. The sterilized film was cut into discs of 10 mm in diameter to fit into the bottom of 48-well tissue culture plates.

### Osteoclast culture

Bone marrow was isolated from the femurs of male Sprague Dawley rats 8 weeks old. Femurs were flushed with sterile PBS using a 5 mL syringe and 25 G needle. Bone marrow was collected in a 15 mL conical tube. Samples were pelleted at 1400 RPM for 5 min, washed once in Dulbecco’s Modified Eagle’s medium (DMEM), resuspended in cryostorage media (90% FBS + 10% DMSO) and stored in liquid nitrogen until use. Experiments were conducted in triplicate (n = 3). For the isolation and differentiation of OSC, the vial of bone marrow was quick thawed at 37^o^C. The bone marrow was collected in complete DMEM (basal media + 10% FBS + 35 μg/mL gentamicin), pelleted to remove residual DMSO, and then resuspended in 10 mL complete DMEM containing 50 ng/mL M-CSF (Peprotech). Cells were plated onto one 10 cm tissue-culture plate. After 72 h, the media was removed and the monolayer was washed with ice cold PBS. This process selected a population of Macrophages from the bone marrow aspirate. Macrophages were lifted using a cell scraper, collected into a tube containing complete media, and spun down at 1000 RPM for 5 min. The pellet was resuspended in complete media containing 50 ng/mL M-CSF, and the volume was split between two 10 cm tissue culture plates. After 48 h, media was changed to complete media containing 50 ng/mL M-CSF and 100 ng/mL RANKL (Peprotech). Cells were cultured for 4 days, with media changes every 48 h. After 4 days of differentiation, mature osteoclasts were lifted from the plate using a cell scraper and seeded onto the various substrates. Cells were cultured in complete media containing 50 ng/mL M-CSF and 100 ng/mL RANKL and allowed to attach for 48 h. Samples were then washed once with PBS and fixed in 4% paraformaldehyde (PFA) for 10 min.

### Osteoclasts imaging

#### Immunostaining

RANK was the chosen selective marker for OSC (see Discussion). Samples were permeabilized with 0.25% Triton-X for 15 min, then blocked in staining solution (4.3% FBS + 1% BSA in 1 × HBSS) for 30 min. Samples were stained with primary antibody, anti-RANK (mouse monoclonal, Life Technologies, 1:100 dilution in staining solution), overnight at 4^o^C. Samples were washed 3× with PBS, then incubated with the secondary antibody, goat anti-mouse 488 (Life Technologies, 1:250 dilution), and rhodamine phalloidin (Life Technologies, 1:500 dilution) in staining solution for 1 h at room temperature. Samples were washed 3× in PBS, then incubated with Hoechst DNA stain (AnaSpec, Inc., 1:500 dilution) in PBS for 10 min at room temperature. Samples were washed 3× in PBS prior to imaging.

#### Fluorescence microscopy

Cells were imaged using an inverted DIC fluorescence microscope (Zeiss Axio Observer D1; Jena, Germany). Images were acquired using the DAPI filter (335–383 excitation, 420–470 emission), Alexa 555 filter (538–562 excitation, 570-40 emission), and GFP filter (450–490 excitation, 500–550 emission) and an AxioCam MRm digital camera. Images were analyzed using NIH ImageJ software.

#### Laser scanning confocal microscopy

Cells were imaged using an inverted laser scanning confocal microscope (Zeiss LSM 780; Jena, Germany). Images were taken using a 63× objective lens (Zeiss, Numerical Aperture 1.4). Images were acquired using a 405-nm laser for DAPI, and a 488-nm laser for Alexa-488, and a 594-nm laser for Alexa-594. Confocal images series (i.e., z-stacks) were taken at increments of 0.7 μm, and a 0.094 micron-to-pixel ratio in lateral resolution. 3D projections of cells were created using Icy (v1.9.4.0, created by the Quantitative Image Analysis Unit at Institut Pasteur), and by using the Stereo 3D Canvas (VTK), which is free and in the public domain [18]. Deconvolution was done using the Zeiss Zen Blue software.

## Results

### Analysis of OSC on spin-coated substrates

**Fig. 4:**
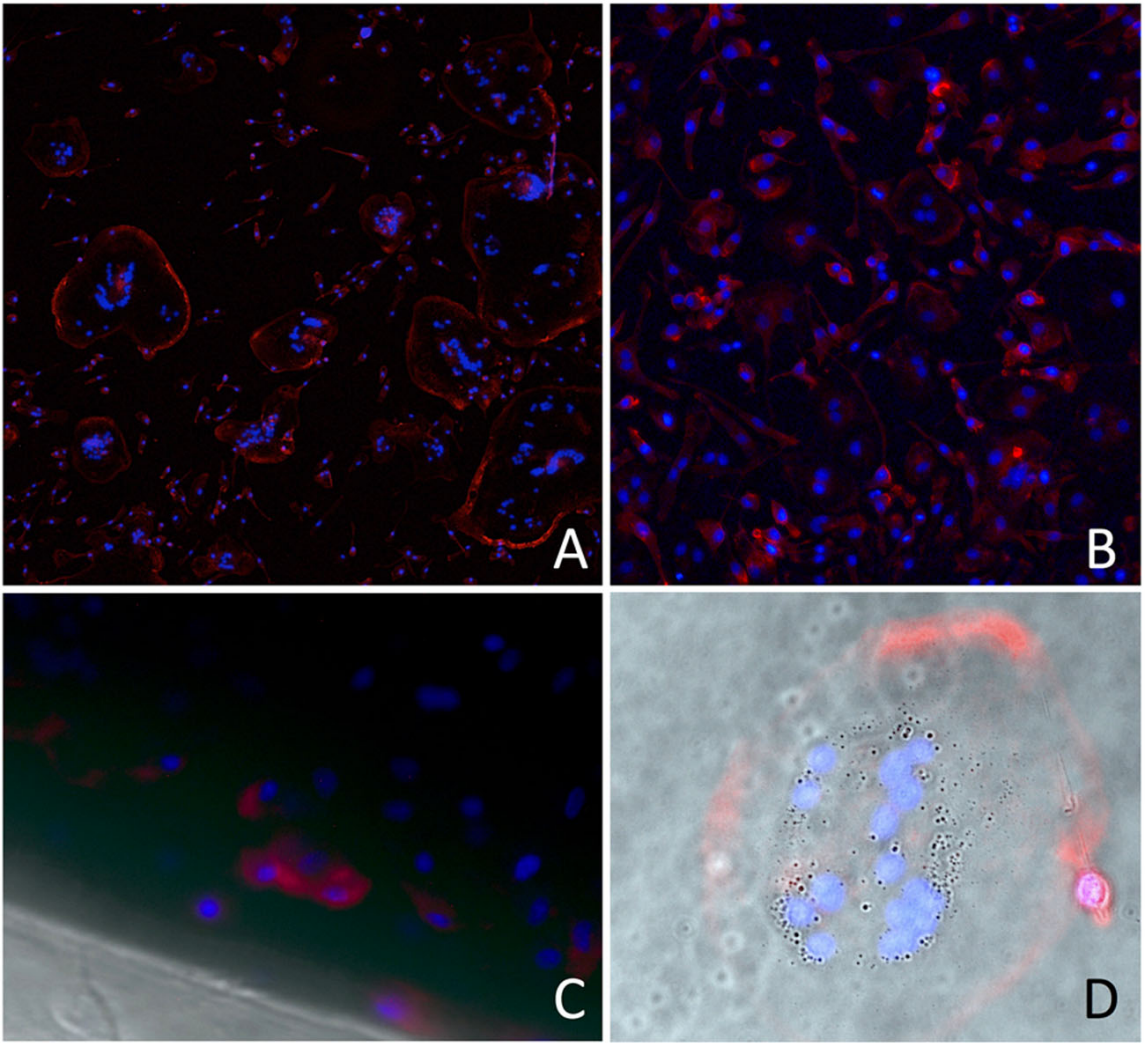
In this serie of images of increasing magnification it is shown that: **a** Actin ring formation is clearly visible on E1001(1k)-RGD (50×) and **b** polystyrene (200×); **c** hydroxyapatite disks are opaque in transmitted light and the signal for Actin and DNA was detectable only at the periphery of the disk (400×); **d** vesicles are visible with phase contrast microscopy within the OSC (E1001(1k)-RGD spin-coated substrate at 630×)

**Fig. 5:**
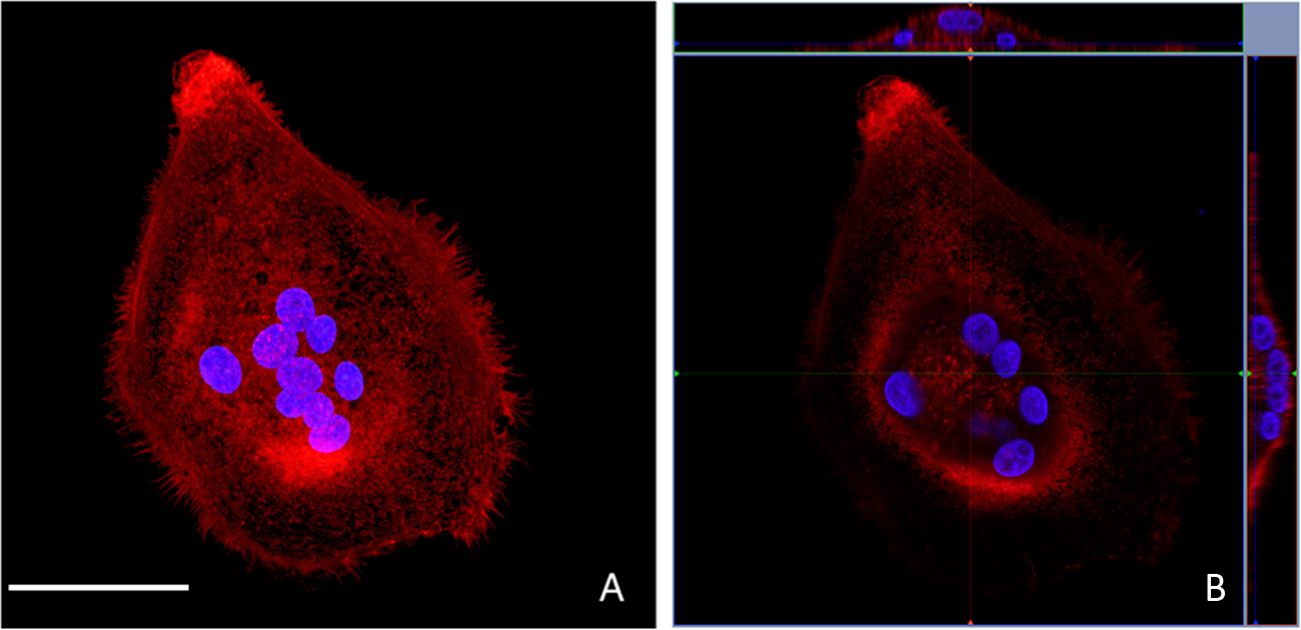
**a** A “maximum intensity projection” gives the reconstruction of multiple transverse confocal sections of an OSC cultured on a spin-coated E1001(1k)-RGD substrate. A high resolution was obtained thanks to the transparency of the substrate. (scale bar = 50 μm) **b** Orthogonal 3D reconstructions centered in the resorption area give views of the dome-shape distribution of nuclei (the blue line indicates the level of the section) (color figure online)

**Fig. 6:**
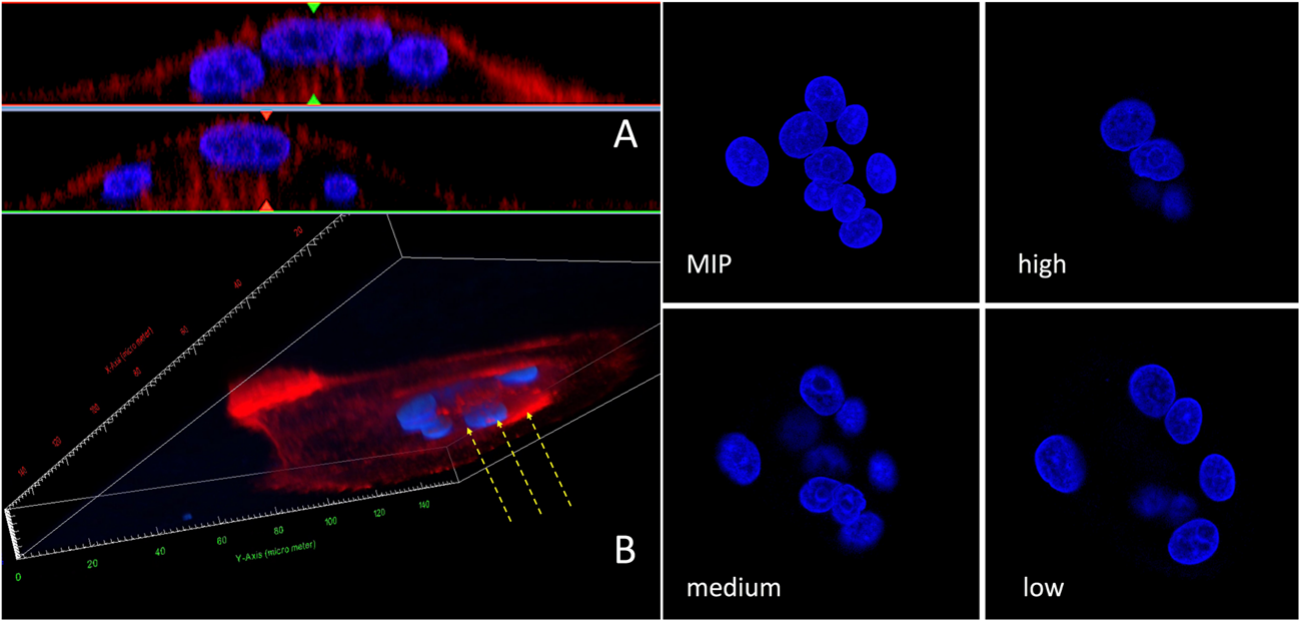
**a** 3D reconstruction visualizes the dome-shaped distribution of nuclei and **b** the presence of actin pillars in the resorption zone (yellow arrows). Maximum intensity projection of isolated nuclei (MIP) and transverse sections taken at three different heights from the substrate, show how the horseshoe arrangement provides space for the actin pillars of the RB (color figure online)

**Fig. 7:**
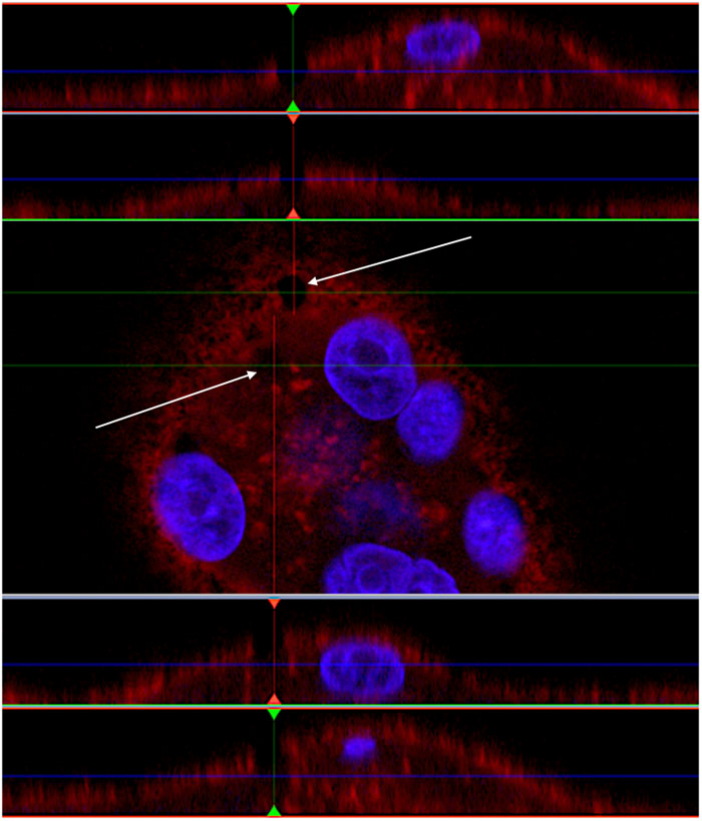
Transparency of the thin spin-coated polymeric substrate allowed the visualization of “channels” that connect the resorption zone to the functional secretory domain (white arrows) (color figure online)

OSC are visible after 48 h of culture on the spin-coated E1001(1k) and E1001(1k)-RGD substrates. Actin appears predominantly at the perimeters, indicative of the formation of the sealing zone. With phase contrast microscopy, vesicles are visible within the OSC. Actin rings are also observed on polystyrene. While polymeric substrates allowed the use of transmitted light microscopy, this was not possible in the majority of the area of the hydroxyapatite disks, but we observed the border of the AR at the periphery of the disk (Fig. 4). 3D reconstruction of laser confocal microscopy images on the 100 nm spin-coated E1001(1k)-RGD polymer film allowed the visualization of actin pillars above the resorption zone; they will constitute the cytoskeleton of the “ruffled border”; however, no resorption was possible on this thin substrate (Fig. 5). Orthogonal 3D reconstructed sections centered in the resorption area give views of the dome-shape distribution of nuclei. 3D visualization of nuclei alone shows how the typical horseshoe arrangement seen close to the substrate provides the space to accommodate the actin pillars of the RB (Fig. 6). Transparency of the substrate allowed for the visualization of “channels” that connect the resorption zone to the functional secretory domain (Fig. 7). At our stage of research it is not possible to consider them as an evidence for the functional “step 3” in OSC activity because no marker was used to detect transcytosed vesicles.

### Analysis of OSC on solvent-casted substrates

**Fig. 8:**
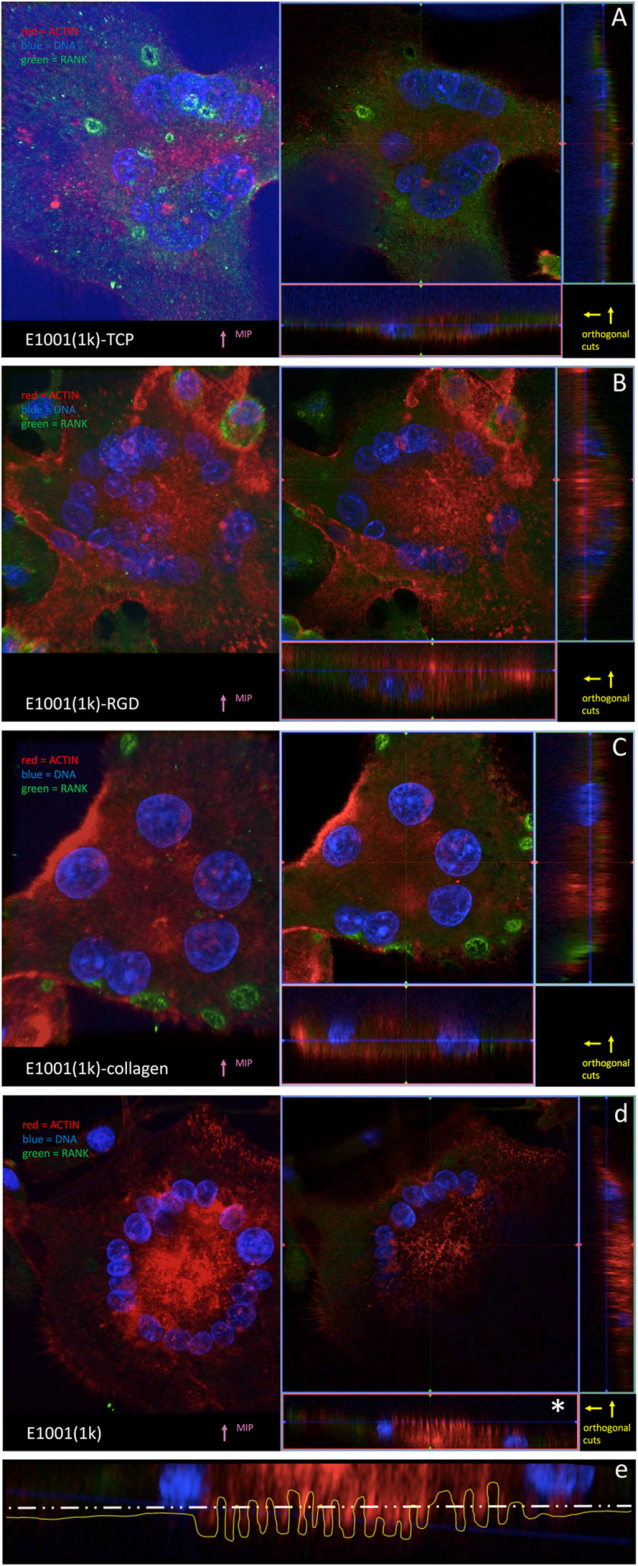
**a** E1001(1k)-TCP imaging was difficult due to the autofluorescence of the calcium phosphate crystals embedded in the substrate, however this clearly helps in visualizing the substrate itself (the “blue” region in the orthogonal sections). **b** E1001(1k)-RGD and **c** E1001(1k)-collagen have a diminished light transmittance through the film, but the actin signal is seen deeper into the substrate below the “drilling complex” region. **d** E1001(1k) is fully transparent and provided higher-definition images where a “ruffled border” interface with the substrate was detectable. **e** An optical section in the “drilling complex” zone (* in **d**) shows a “ruffled border” profile of the actin pillars (yellow dotted line; the white line represents the substrate profile; the blue line represent the confocal transverse section) (color figure online)

Thicker solvent-casted films provided a substrate that can be resorbed. The transparency of the solvent-casted E1001(1k) alone film enabled high resolution imaging. The incorporation of RGD and collagen I into the polymer film decreased the degree of light transmittance through the film, thus reducing the image resolution. Imaging of the E1001(1k)/CaP substrate was even more difficult due to the autofluorescence of the large calcium phosphate crystals embedded in the substrate. However, AR formation was documented on all the substrates. E1001(1k) showed the highest number of cells with typical morphological characterstics of osteoclasts, like a periphery of filopodia and a circular or horseshoe arrangement of nuclei around the “drilling complex”. 3D reconstruction and orthogonal sections showed that the actin fluorescence signal can be detected into the surface of the substrate, but only below the drilling complex. Since the quality of imaging varied from substrate to substrate, the best resolution was obtained with the full transparent E1001(1k). For this reason, E1001(1k) allowed the repeated visualization of a structured border below the drilling complex which presents the morphological characters of the “ruffled border” membrane (Fig. 8).

## Discussion

The idea to have osteoclasts (osteo-**c**-lasts) to resorb the bone graft material and to direct new bone formation and vascularization is based on the principles of bone biology. At the same time, this idea is a leap forward in a research field which has focused, in the past three decades, mostly on supporting osteoblasts (osteo-**b**-lasts) and on inducing bone formation, without recognizing the role of osteoclasts and the importance of bone formation obtained following bone resorption [19]. In a similar approach, the promotion of osteoclastogenesis and angiogenesis has been attempted to integrate and remodel bone allografts [20–23]. A clinically-effective bone graft material able to address major bone loss will be regarded as a real clinical advancement. Current materials (often called “artificial bone substitutes”) promote the early formation of bone, but fail to complete the healing process in large defects [1, 2]. The inability to achieve the “macroscopic bone remodeling” (the process by which bone restructures continuously to respond to external mechanical loads [24]) may be due to a failure to re-establish the “microscopic bone turnover”.

The introduction of “artificial bone substitutes” in clinical use generated great expectations in surgeons whose goal was to overcome the limitations of autografts (demanding surgical technique; limited availability; donor-site morbidity) and of allografts (inability to achieve a full integration with host bone; variability in the quality of bone because of factors associated with the donor) [25]. In most cases, bone formation is limited in space and time [20, 26]. Quite interestingly to us, allografts and non-vascularized autografts may not elicit an adequate healing response if bone remodeling is limited and new blood vessels do not extend the host’s vascular network into the graft [25]. From the material point of view this seems to be odd, as both allografts and non-vascularized autografts have a composition and structure that matches healthy bone in a far better way than any engineered artificial bone substitute. We speculate that factors that trigger BTO may have been lost in dead allografts or in hypoxic non-vascularized autografts. Therefore, the understanding of the basic process and regulation of BTO becomes critical to identify the key players in bone regeneration.

The fact that the CaP-free E1001(1k) is a suitable substrate for osteoclast culture was an unexpected finding. CaP-free polymer formulations, like poly-L-Lactic Acid (PLLA), have not been shown to support OSC differentiation [27]. However, this makes E1001(1k) a promising candidate in the pursuit of a biomaterial that recruits osteoclasts to re-establishes BTO. The creation of a less acidic environment, in comparison with most of the clinically-used degradable biomaterials like PLLA, may be a clue for an OSC supporting material as OSC function will be limited in an acidic environment.

As we found that OSC can be cultured on all the E1001(1k) substrates, our attention was however quickly re-focused on E1001(1k) alone, because the unmodified polymer has the advantage of being an easier-to-produce material in comparison with its blends and because of its better optical properties. Transparency is an additional unexpected benefit of E1001(1k) and it allowed the visualization of key morphological features of osteoclasts (actin ring, multiple nuclei, expression of RANK, ruffled border) by transmitted light (instead of reflected light). It also avoided the need to transversely cut the sample as it would be required by transmission electron microscopy and focused ion-beam dissection [28]. The quality in the visualization of the interface between osteoclast and substrate and the avoidance of the destructive cut of the sample (both allowed by transmitted light) may promote a new *in vitro* modality in the study of osteoclasts behavior and their response to drugs supposed to regulate their activity.

The search for evidences of a possible transcytosis activity is one of our future goals. It is known that the transcytotic pathway is supported by thick bundles of microtubules which span from the RB tyo the FSD [8]. Theoretical speculation suggests that both collagen and needle-shaped hydroxyapatite crystals should be incorporated in the our polymer for transcytosis to occur; however, we would like to explore the role of the actin channels evidenced in Fig. 7 when these components where not present into the substrate. Another future goal is the better differentiation between true OSC and morphologically similar MNGC (like those found in a foreign body reaction, for example). OSC themselves are a subset of MNGC, and while they share the same precursor cells, OSC reside specifically in the vicinity of bone, whereas MNGC are found at pathological sites of inflammation and surfaces of foreign bodies. OSC are capable of resorbing bone completely, whereas MNGC are only capable of dissolving the mineral phase. While both cell types are capable of forming an actin ring, MNGC are unable to digest the matrix of the bone due to the inhability to form a RB and to express cathepsin K [29]. Calcitonin receptor and RANK are considered as the specific markers for OSC while MNGC exclusively express CD86 and HLA-DR [30]. While in this study we have demonstrated positive staining of RANK in our OSC, we plan to add specific MNGC markers as a control. We advocate that a similar procedure should be adopted in the study of OSC because the traditional OSC marker, the tartrate resistant acid phosphatase (TRAP), can stain both types of cells (even if affinity for OSC is greater [19]).

## Conclusions

This is the first report of a high resolution imaging in visible light of the OSC “actin ring” and “ruffled border” formation on CaP-free polymeric bone graft material. The polymer E1001(1k) supports OSC in-vitro culture and its tranparency allows the visualization of key morphological features without the need to destructively cut the cell. This may lead to the development of an analytical method which may provide the same information as transmission electron microscopy but will not require the complex sample preparation prior to imaging. Furthermore, the sample will remain intact for subsequent analysis. We are aware that this initial study is just the very first step in a proposed new strategy for bone grafting based on the recruitment of OSC to re-establish BTO and its concurrent new vascularized bone formation. However, our initial results unveiled new possibilities for the in-vitro study of OSC and this may be beneficial in other applications as well.
